# Surface Doping of Organic Single‐Crystal Semiconductors to Produce Strain‐Sensitive Conductive Nanosheets

**DOI:** 10.1002/advs.202002065

**Published:** 2020-12-18

**Authors:** Shun Watanabe, Ryohei Hakamatani, Keita Yaegashi, Yu Yamashita, Han Nozawa, Mari Sasaki, Shohei Kumagai, Toshihiro Okamoto, Cindy G. Tang, Lay‐Lay Chua, Peter K. H. Ho, Jun Takeya

**Affiliations:** ^1^ Material Innovation Research Center (MIRC) and Department of Advanced Materials Science Graduate School of Frontier Sciences The University of Tokyo 5‐1‐5 Kashiwanoha Kashiwa Chiba 277‐8561 Japan; ^2^ AIST‐UTokyo Advanced Operando‐Mesurement Technology Open Innovation Laboratory (OPERANDO‐OIL) AIST 5‐1‐5 Kashiwanoha Kashiwa Chiba 277‐8561 Japan; ^3^ Precursory Research for Embryonic Science and Technology (PRESTO) 4‐1‐8 Honcho Kawaguchi Saitama 332‐0012 Japan; ^4^ International Center of Materials Nanoarchitectonics (WPI‐MANA) National Institute for Materials Science (NIMS) 1‐1 Namiki Tsukuba 305‐0044 Japan; ^5^ PI‐CRYSTAL Inc. 5‐4‐19 Kashiwanoha Kashiwa Chiba 277‐0882 Japan; ^6^ Department of Physics National University of Singapore Lower Kent Ridge Road Singapore S117550 Singapore; ^7^ Department of Chemistry National University of Singapore Lower Kent Ridge Road Singapore S1175502 Singapore

**Keywords:** 2D electronic system, molecular doping, organic semiconductors, piezoresistive effect, single crystals

## Abstract

A highly periodic electrostatic potential, even though established in van der Waals bonded organic crystals, is essential for the realization of a coherent band electron system. While impurity doping is an effective chemical operation that can precisely tune the energy of an electronic system, it always faces an unavoidable difficulty in molecular crystals because the introduction of a relatively high density of dopants inevitably destroys the highly ordered molecular framework. In striking contrast, a versatile strategy is presented to create coherent 2D electronic carriers at the surface of organic semiconductor crystals with their precise molecular structures preserved perfectly. The formation of an assembly of redox‐active molecular dopants via a simple one‐shot solution process on a molecularly flat crystalline surface allows efficient chemical doping and results in a relatively high carrier density of 10^13^ cm^−2^ at room temperature. Structural and magnetotransport analyses comprehensively reveal that excellent carrier transport and piezoresistive effects can be obtained that are similar to those in bulk crystals.

## Graphical abstract

The surface of organic single crystals is chemically doped via a simple one‐shot solution process, and the resulting 2D electronic system with a high carrier density serves as a multi‐functional, conductive nanosheet.

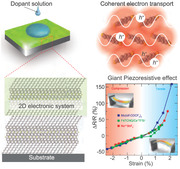

## 1 Introduction

Impurity doping of semiconductors has given rise to electronic devices that have become essential for human life.^[^ ^1^, ^2^ ^]^ A dopant, that is, either an electron donor or acceptor, is implanted into a solid‐state semiconductor and is capable of tuning the Fermi energy.^[^ ^1^, ^2^ ^]^ The concept of impurity doping established in current silicon electronics has been extended to organic semiconductors (OSCs), where molecular dopants rather than atomic dopants are required for redox reactions.^[^ ^3^, ^4^, ^5^, ^6^ ^]^ Various dopants and processes have been utilized to achieve efficient chemical doping of OSCs, particularly for p‐type doping. Unlike impurity doping of inorganic semiconductors, where an impurity dopant is implanted atomistically into a periodic covalent crystal, molecular doping of organic semiconductors involves an admixture of dissimilar molecules, and this is referred to as a host–guest molecular system. Electron transfer occurs between a semiconductor and a dopant following a one‐to‐one redox reaction; therefore, molecular doping in OSCs requires a delicate balance between a high redox potential and appropriate structural hybridization. Recent developments in molecular doping have realized highly efficient doping, particularly in polymeric semiconductors, where new routes such as photo‐assisted doping,^[^ ^7^ ^]^ self‐compensation by covalently bonded counter‐ions,^[^ ^8^, ^9^ ^]^ and anion‐exchange doping^[^ ^10^, ^11^ ^]^ have been proposed. n‐Type doping has also been demonstrated with latent anion donors in self‐compensate system.^[^ ^12^ ^]^ However, it is controversial whether or not the concept of molecular doping can be extended to single‐crystal, small molecular semiconductors, and it is more reasonable to envisage that molecular doping would be less beneficial because the introduction of a relatively high density of dopants inevitably destroys the highly established framework of molecular arrangement present when the crystal structure is that of a purely single molecular species.

With the vast array of recent developments in printing technologies for single‐crystal forms of small molecule OSCs,^[^ ^13^, ^14^, ^15^, ^16^, ^17^, ^18^ ^]^ as well as those in materials science,^[^ ^19^, ^20^, ^21^ ^]^ the production of flexible, multi‐functional electronic devices is expected to be within reach. Various groups have recently developed state‐of‐the‐art OSCs, and the electronic properties of organic devices have been considerably improved as the mobility exceeds 10 cm^2^ V^−1^ s^−1^.^[^ ^22^, ^23^, ^24^, ^25^, ^26^ ^]^ Together with inventions in the printing field, highly reliable, reproducible transistor arrays can be manufactured to produce high‐intensity integrated circuits with relatively high operation speeds up to a few tens of megahertz.^[^ ^27^ ^]^ The excellent electronic properties of single‐crystalline OSCs originate from the chemical structure of designed molecules^[^ ^20^, ^21^, ^24^ ^]^ and the highly periodic electrostatic potential, even though established in van der Waals bonded organic crystals, and are thus a foundation for the realization of coherent, band electron systems where the electron wavefunction is delocalized over molecular crystals^[^ ^28^, ^29^, ^30^ ^]^; therefore, it is essential to control the electronic system while preserving the precisely designed crystal structure. While impurity doping is an effective chemical process for precisely tuning the energy of electronic systems, it always faces an unavoidable difficulty in molecular crystals because the introduction of a high density of dopants inevitably destroys the highly ordered molecular arrangement.^[^ ^4^, ^5^, ^31^ ^]^ Various attempts have been made in the p‐type doping of bulk molecular crystals, which has resulted in either unintentional structural hybridization or has been ineffective.^[^ ^4^, ^5^, ^31^, ^32^ ^]^ As an alternative, interfacial engineering that uses a donor–acceptor interface has been attempted in order to realize controllable interfacial electronic systems.^[^ ^33^, ^34^, ^35^, ^36^, ^37^, ^38^ ^]^ However, little is known to date as to how a delicate balance can be established in a hybrid structure, and whether coherent electron conduction can be preserved after doping.

In striking contrast to previous molecular doping that has been achieved through bulk intercalation of dopants into a host semiconductor, we demonstrate a static method to produce band‐like 2D electronic carriers on the surface of organic semiconductor crystals with their molecular structure preserved. Molecular dopants are deposited gently via a solution process onto a molecularly flat crystallized surface composed of an assembly of small‐molecule semiconductors, which prevents damage to the crystal structure. X‐ray diffraction measurements conclude that the original highly‐crystalline structure is preserved after doping. Observations of the Hall effect and excellent piezoresistive effects evidence intermolecular charge coherence for the carriers in the conducting nanosheet at the crystalline surface. We also successfully implement surface‐doped OSC thin film in a high‐sensitivity strain sensor. The present doping method and the variation of dopants are useful tools for creating highly conducting 2D electronic systems.

## 2 Results and Discussion

### 2.1 Surface Doping of Single‐Crystal OSCs and Characterization of Doping Effects

**Figure 1:**
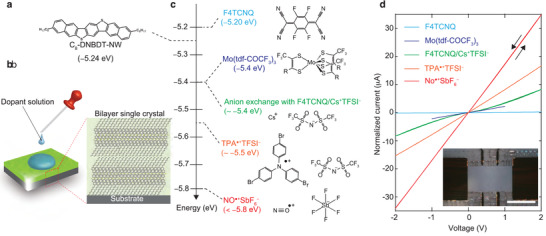
Concept of surface doping of single crystal OSC C_8_–DNBDT. a) Molecular structure of C_8_–DNBDT. Single‐crystal bilayer films of C_8_–DNBDT were fabricated by the continuous edge‐casting method. b) Doping was performed simply by exposing solid‐state thin films of bilayer C_8_–DNBDT to a solution of the dopant. The excess solution was then removed by spinning the substrate. c) Molecular structure and electron affinity (*E*_a_) of the dopants used in this work. *E*_a_ was estimated by photoemission yield spectroscopy measurements with PBTTT as a reference semiconductor host (see Experimental Section). d) Current–voltage (*I*–*V*) characteristics of doped C_8_–DNBDT. The obtained current was normalized with respect to the length and width so that data for all five samples could be displayed consistently. Four‐terminal conductivity was derived from the *I*–*V* characteristics. The inset shows a micrograph of the fabricated device (the anion‐exchange doping sample). The scale bar represents 200 µm.

As an ideal test subject to investigate the surface doping of single‐crystalline semiconductors, our benchmarked OSC, 3,11‐dioctyldinaphtho[2,3‐*d*:2',3'‐*d*']benzo[1,2‐*b*:4,5‐*b*']dithiophene (C_8_–DNBDT; molecular structure shown in **Figure** **1**a) was deposited using continuous edge casting, which is a meniscus‐guided coating (MGC) technique.^[^ ^13^, ^14^, ^15^, ^16^, ^17^, ^18^, ^39^ ^]^ In continuous edge casting, a self‐assembled molecular nanosheet grows at the vapor–liquid interface and is then laminated onto a substrate.^[^ ^26^ ^]^ Bilayer single‐crystalline thin films with a molecularly flat surface were prepared selectively by adjustment of the substrate temperature and the solubility.^[^ ^22^, ^23^, ^25^, ^26^ ^]^ Note that single‐crystalline thin films used for transport and X‐ray diffraction measurements were true mono‐domain crystals with no grain boundaries.^[^ ^22^, ^25^, ^26^ ^]^ Chemical doping (impurity doping) was performed simply by exposing solid‐state thin films of C_8_–DNBDT to a solution of the dopant (Figure 1b). Five different electron acceptor dopants were used: F4TCNQ, Mo(tfd‐COCF_3_)_3_, F4TCNQ/[Cs^+^TFSI^−^] (anion exchange), [TPA^• +^TFSI^−^], and [NO^• +^SbF_6_^−^], where F4TCNQ represents tetrafluorotetracyanoquinodimethane, Mo(tfd‐COCF_3_)_3_represents molybdenum tris(1‐(trifluoroacetyl)‐2‐(trifluoromethyl)ethane‐1,2‐dithiolene), TFSI represents bis(trifluoromethylsulfonyl)imide, TPA represents tris(4‐bromophenyl)aminium, and SbF_6_^−^represents hexafluoroantimonate). The molecular structures of the dopants are shown in Figure 1c. For F4TCNQ and Mo(tfd‐COCF_3_)_3_, one‐electron transfer occurs between C_8_–DNBDT and these dopants, for example, C_8_–DNBDT + F4TCNQ → [C_8_–DNBDT^• +^F4TCNQ^•^].^[^ ^4^, ^5^, ^40^, ^41^ ^]^ Molecular doping through an anion exchange process, as reported recently,^[^ ^10^ ^]^ was also used, where the initial electron transfer was initiated by F4TCNQ, after which the F4TCNQ anion is spontaneously exchanged with the second anion (TFSI^−^) supplied from a large excess of [Cs^+^TFSI^−^] salt dissolved in the solvent. In addition, strong molecular oxidants TPA^• +^and NO^• +^ were employed. Redox reaction occurs between C_8_–DNBDT and these oxidants, for example, C_8_–DNBDT +TPA^• +^ → C_8_–DNBDT^• +^+ TPA. To satisfy the charge neutrality in the resultant thin film, a C_8_–DNBDT cation forms a donor–acceptor pair with a counter‐anion of these oxidants (TFSI^−^and SbF_6_^−^); C_8_–DNBDT +[TPA^• +^ TFSI^−^]→ [C_8_–DNBDT^• +^TFSI^−^] + TPA. **Table** **1** summarizes the conditions for chemical doping used in this work. Note that during the immersion process, solvents that are orthogonal to C_8_–DNBDT were used.

**Table 1:** Details of the process conditions for the chemical doping methods used in this work

Dopants	Conductivity^a)^ [*μ*S]	Solvent	Concentration [wt%]	Process temp. [°C]	Process time [s]
F4TCNQ	0.1	*n*BA	0.3	r.t.	60
Mo(tfd‐COCF_3_)_3_	2	CT‐Solv.180	0.05^b^	60	900
F4TCNQ/Cs^+^TFSI^−^	4	nBA	0.3 /0.3	r.t.	30
TPA^• +^TFSI^−^	8	ACN	0.1	60	30
*p*‐mTFF‐F3TSFI^c^	11.5	ACN	0.8	40	10
NO^• +^SbF_6_^−^	17	ACN	0.05	r.t.	10

^a)^Four‐terminal sheet conductivity *σ*_□_, was measured using the Hall bar geometry; ^b)^Although the solubility of Mo(tfd‐COCF_3_)_3_ in CT‐Solv.180 was very poor, a suspension was used, as described in ref. [41]; ^c)^ *p*‐mTFF‐F3TSFI denotes doped poly(9,9‐bis(3‐(trifluoromethanesulfonylimidosulfonyl)‐propyl)fluorene‐2,7‐diyl‐alt‐1,4‐phenylene‐(m‐trifluoromethylphenylimino)‐1,4‐phenylene).^[^ ^8^, ^9^ ^]^ *n*BA, *n*‐butyl acetate; ACN, acetonitrile.

To assess the degree of doping, the four‐terminal sheet conductivity (*σ*_□_) was measured using the Hall bar geometry (Figure 1d, and the results are summarized in Table 1). *σ*_□_ for the C_8_–DNBDT film anion‐exchanged with [Cs^+^TFSI^−^] was increased to 4 *μ*S, which is significantly higher than that for those doped solely with F4TCNQ and Mo(tfd‐COCF_3_)_3_. The maximum *σ*_□_ was 17 *μ*S in the C_8_–DNBDT films doped with [NO^• +^SbF_6_^−^], which is comparably high and similar to those for rubrene doped with fluorinated alkylsilanes (10 *μ*S),^[^ ^34^ ^]^ and approximately threefold higher than the best possible conductivity achieved in field‐effect transistors.^[^ ^30^ ^]^

The capability of chemical doping can be determined primarily by the electrochemical redox potential between the host semiconductor and the dopant. This simplistic Marcus theory is used to explain host–guest binary systems.^[^ ^10^ ^]^ However, an additional energy gain from anion exchange is obtained for anion exchange doping, which further promotes the doping level.^[^ ^10^ ^]^ To scale the capability of doping for the five dopants, the energy levels (relative to the reference p‐type polymer) are plotted in Figure 1c.^[^ ^10^, ^40^, ^41^, ^42^ ^]^ In principle, these energies are identical to the electron affinity of the dopants, except for anion exchange doping with F4TCNQ/[Cs^+^TFSI^−^]. The degree of doping is correlated with the energy level, that is, a higher electron affinity of the dopant species gives rises to more efficient electron transfer to C_8_–DNBDT, resulting in higher conductivity.

**Figure 2:**
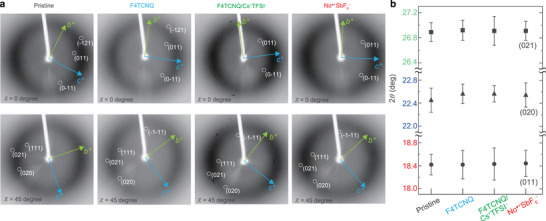
Single‐crystal XRD measurements of C_8_–DNBDT thin films. a) Raw transmission XRD patterns collected with an X‐ray incident angle almost perpendicular to the substrate plane. The angle *χ* in the top and bottom panels defines the rotation of the sample around its surface normal. Samples from left to right were pristine (undoped), doped with F4TCNQ, with F4TCNQ/Cs^+^TFSI^−^(anion exchange), and with NO^• +^SbF_6_^−^. b) Diffraction spots, indicated by 2*θ* with respect to the different Miller indices (squares: (021); triangles: (020); circles: (011)). Error bars indicate one standard deviation.

**Figure** **2**a shows single‐crystal X‐ray diffraction (XRD) patterns for C_8_–DNBDT thin films (pristine, doped with F4TCNQ, anion‐exchanged with Cs^+^TFSI^−^, and NO^• +^SbF_6_^−^; from left to right). Transmission XRD patterns were collected with X‐ray incidence almost perpendicular to the substrate plane, so that in‐plane diffraction was collected on the imaging plate. The angle *χ* in the top and bottom panels denotes the rotation of the sample around its surface normal. The diffraction spots, indicated by 2*θ* with respect to the different Miller indices (squares: (021); triangles: (020); circles: (011) in Figure 2b) were almost the same, regardless of the dopant. Diffraction peaks in transmission XRD measurements generally arise from the entire bulk of the sample, and the surface structure cannot be analyzed separately. On the other hand, a bilayer of C_8_–DNBDT with a thickness of ≈8 nm was deposited and investigated selectively; therefore, the peaks obtained should be sensitive to changes in the surface structure. XRD measurements obtained for the present bilayer films indicated that the original crystal structure of the bilayer C_8_–DNBDT does not change after the chemical doping procedure. This is in clear contrast to chemical doping in polymeric semiconductors, where dopants intercalate throughout the bulk polymer network and reside at particular side‐chain regions.^[^ ^10^, ^40^, ^41^ ^]^ However, the high‐energy barrier in molecular crystals can prevent dopant intercalation. It is more reasonable to consider that a single crystal with dense molecular packing does not possess a free internal volume to permit the intercalation of dopants. Note that the *π*‐conjugated cores in C_8_–DNBDT assemble themselves into a herringbone packing arrangement across the in‐plane direction (*b*‐ and *c*‐axes), whereas a layer‐by‐layer stack is realized along the out‐of‐plane direction (*a*‐axis). It is also unrealistic to suppose that dopants intercalate between the layers. Density functional theory calculations estimated that the energy to separate the vertically‐stacked layers was approximately 0.5 eV, which is very high compared to the energy gain to form a donor‐acceptor pair.^[^ ^26^ ^]^ Surface doping effects using polymeric dopants were also examined. Self‐compensated, heavily doped polymers exhibit excellent hole doping capability to adjacent OSC molecules.^[^ ^8^, ^9^ ^]^ A relatively high *σ*_□_ of 11.5 *μ*S was obtained for C_8_–DNBDT doped with self‐compensated triarylamine–fluorene copolymers with tethered trifluoromethylsulfonylimide counter anions (*p*‐mTFF‐F3TSFI). The tethered anions are covalently‐bounded to the polymer main chain; therefore, the counter anion, in principle, do not intercalate into the adjacent OSC molecules. The observation of scalable *σ*_□_ with electron affinity obtained for the polymer dopants (the electron affinity was measured to be up to 5.6 eV for self‐compensated polymers^[^ ^8^, ^9^ ^]^) provides further evidence that a high carrier‐density nanosheet is realized at the surface of C_8_–DNBDT. Overall, it is concluded that no bulk intercalation of dopants occurs in the bilayer of C_8_–DNBDT, that is, the electrical conductivity arises from the single‐crystal surface‐doped region C_8_–DNBDT. Although the proof‐of‐concept of surface doping is demonstrated in this work, further spectroscopic investigations will be needed to clarify the surface electronic state in future.

### 2.2 Coherent Band‐Like Carrier Transport and Piezoresistive Effect

**Figure 3:**
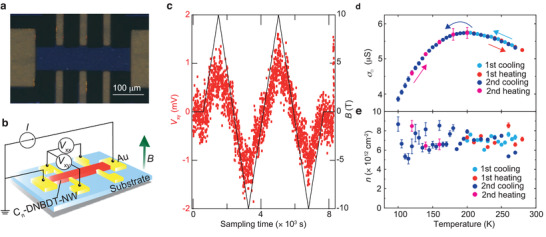
a) Photograph and b) schematic illustration of fabricated Hall bar for anion‐exchange doped C_8_–DNBDT thin film. The longitudinal *V*_xx_ and transverse *V*_xy_ electromotive forces were monitored simultaneously under application of a constant dc current, *I* = 1 *μ*A. c) An external magnetic field *B* was ramped up to 10 T and down to −10 T (right axis). Transverse voltage *V*_xy_ measured at 210 K (left axis). d) Four‐terminal sheet conductivity *σ*_□_ as a function of temperature. e) Sheet carrier density *n* derived from the inverse Hall coefficient (*n* = *eR*_H_)^−1^ as a function of temperature. The Hall effect measurements were performed with cooling and heating cycles (1st cooling from 280 to 200 K, 1st heating from 200 to 270 K, 2nd cooling from 270 to 100 K, and 2nd heating from 100 to 200 K.) Note that no apparent *V*_xy_ drift with respect to sampling time was confirmed during the entire temperature dependence measurements (approximately 200 h).

We now consider the charge transport properties of a highly conductive surface. Hall effect measurements are a powerful method not only to determine the delocalized carrier density and the Hall mobility, but also to distinguish the nature of electron localization.^[^ ^28^, ^29^, ^30^ ^]^ The Hall voltage should be observable only when the charge carriers undergo band transport, that is, when electron conduction can be described using the Boltzmann transport framework. The Hall measurements were performed using a standard Hall bar geometry for the anion‐exchanged sample (the doped state consists of donor‐acceptor pairs [C_8_–DNBDT^• +^TFSI^−^]), as shown in **Figure** **3**a,b, where the longitudinal voltage *V*_xx_ and transverse voltage *V*_xy_ were monitored while applying a constant dc current *I* of 1 *μ*A and ramping the external magnetic field, *B*. A clear transverse voltage was observed (a typical *V*_xy_ response to *B* at temperature *T* = 210 K is shown in Figure 3c). The sign of the Hall voltage is consistent with hole conduction. These results indicate that charge carriers at the surface doped state in C_8_–DNBDT undergo coherent band transport. In addition to the Hall effect measurements, coherent band‐like transport is also evidenced by the temperature dependence of *σ*_□_ (Figure 3d). The measured *σ*_□_  increases with decreasing *T*. Below *T* = 200 K, *σ*_□_ decreases weakly because the carriers are likely to be captured by shallow trap states, which is consistent with the trend observed in the previous study.^[^ ^30^ ^]^ We do not provide details of the charge transport mechanism here; however, we would like to emphasize that 2D coherent transport can be realized in the surface‐doped C_8_–DNBDT nanosheet, as long as the carriers are free from these trap states at room temperature. Note that molecular steps that appear at the surface of crystalline OSCs are likely to capture charge carriers, which does result in limitations of carrier mobility to some extent.^[^ ^43^ ^]^ In contrast, no apparent crystalline steps were observed for the surface doped C_8_–DNBDT, presumably because the bilayer nature of the C_8_–DNBDT thin films without molecular steps may provide an ideal flat surface.

The sheet carrier density *n*_□_, derived from the inverse Hall coefficient, *n*_□_  = (*eR*_H_)^−1^, was measured to be 7 × 10^12^, and was almost independent of the temperature (Figure 3e). These values are approximately 2–3 times larger than those achieved using field‐effect transistors.^[^ ^30^ ^]^ The excellent charge transport properties realized at the surface of the C_8_–DNBDT nanosheet are attributed to the preservation of the periodic crystal potential that is designed originally to have an efficient overlap of transfer integrals between neighboring molecules, that is, the effective mass. The original herringbone crystal packing structure in C_8_–DNBDT is not disturbed by the present doping process; therefore, the initially‐designed electron conduction can be maintained, even after the chemical doping processes. This is in contrast to bulk doping in single‐crystalline OSCs.^[^ ^4^, ^5^, ^31^, ^32^ ^]^ Previous studies have attempted to chemically dope bulk organic single crystals, for example, by the homo‐epitaxial growth of a host OSC and dopant,^[^ ^32^ ^]^ and by electrochemical oxidation.^[^ ^44^ ^]^ These approaches were successful in realizing a relatively high bulk electrical conductivity, but only when the resultant crystal packing structure was predictable and suitable for charge transfer and propagation of the electron wavefunction.

**Figure 4:**
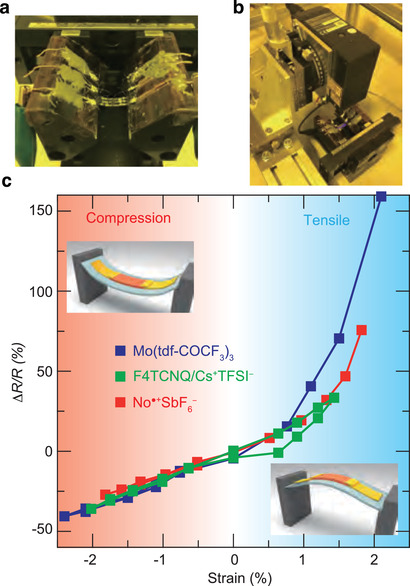
Strain effect on surface doped C_8_–DNBDT thin films. a) Photograph of *I*–*V* measurement setup under compressive and tensile strains. b) The strain that was applied by bending the thin films was monitored by a laser displacement meter. c) Changes in resistance $\frac{\Delta R}{R}$ as a function of applied strain. Negative strain indicates compressive strain and vice versa.

We present further evidence that the 2D electron system realized by surface doping preserves the excellent functionality of the original crystal structure. As observed in many condensed matters, the electronic conduction in OSCs can be modulated by an external force.^[^ ^45^, ^46^, ^47^ ^]^ It was supposed that uniform compression of the crystal structure would directly suppress the vibration of molecules, which would increase the momentum relaxation time. We do not attempt to investigate the detailed mechanism of the unique piezoresistive effect in OSCs, but rather present how surface doping has an impact on the magnitude of the strain effect. **Figure** **4** shows the strain effect on the surface doped C_8_–DNBDT thin films. Changes in the four‐terminal resistance Δ*R*/*R* were measured under compressive or tensile strain using an in‐house‐built experimental setup (Figure 4a), and the surface strain ϵ was determined directly from measurement of the surface curvature radius *r* using a laser displacement meter (Figure 4b) and the substrate thickness *h*_s_ (ϵ =$\frac{h_{s}}{2r}\times 100$). Here, the strain was applied along the *c*‐axis, which is the transport axis. Similar to the XRD and conductivity measurements, a mono‐domain single crystal of bilayer C_8_–DNBDT thin film was employed to characterize the strain effect.

Figure 4c shows the changes in Δ*R*/*R* under compressive (ϵ < 0) and tensile strain (ϵ > 0) for three different samples (doped with Mo(tfd‐COCF_3_)_3_, F4TCNQ/[Cs^+^TFSI^−^], and NO^• +^SbF_6_^−^). A positive piezoresistive effect, where the resistance decreases under compressive strain, was measured for all three samples and was independent of the dopant employed. Note that the measured resistance recovered to the original value after multiple applications of compressive and tensile strain, that is, there was no significant hysteresis observed for ϵ being 0 → −2.5 → 0 → +1 → 0 → −2.5 → 0%, etc. On the other hand, Δ*R*/*R* was unstable when a relatively large tensile strain of ϵ ≈ 2% was applied. This may be because uncontrollable cracks and/or defects were generated in the single‐crystalline thin films as a result of the tensile strain. Therefore, we focused on the strain effect for relatively narrow strain ranges. The gauge factor (GF), which is the index of the relative change in electrical resistance under mechanical strain, was estimated to be 17 ± 0.2 (from the slope of Δ*R*/*R* vs ϵ), which is consistent with that reported previously.^[^ ^45^ ^]^ A relatively large piezoresistive effect was reported for a similar DNBDT analog with field‐effect transistor geometry, that is, the resistance of the single‐crystalline DNBDT thin film was improved by 60% under 3% compressive strain. For a single‐crystalline OSC, the mechanical strain is applied not to the grain boundaries, but to the crystal lattice; therefore, the unique strain effect, particularly in organic single crystals, originates purely from the intrinsic molecular assembly. The consistency of the GF obtained through two different doping methods (chemical vs field‐effect doping) indicates that the electron conduction induced by the present mild doping results in excellent functionality derived from the original crystal structure. Note that the obtained GF of approximately 17 for single crystals of OSCs is significantly larger than that for metals. The piezoresistive effect in a typical isotropic metal arises from the change of geometry that results from applied mechanical strain, where the GF expected from the Poisson ratio of metals is approximately 2.^[^ ^48^ ^]^

The dopant agents used were compared with respect to the stability of the doped state. Conventional molecular doping with molecular oxidants such as F4TCNQ and Mo(tfd‐COCF_3_)_3_ facilitates a half‐cell redox reaction, which gives rise to one‐electron transfer in the ground state. The redox reaction PBTTT + F4TCNQ ⇌ [PBTTT^• +^F4TCNQ^−^] has a finite equilibrium rate constant, that is, a charge back‐transfer occurs with a particular rate constant. The population of doped states [PBTTT^• +^F4TCNQ^−^] in the ground state can fluctuate due to the back‐transfer; therefore, the resultant doping level, in principle, varies according to the degree of back‐transfer. In addition, a loss of doping occurs when dopants are volatile. There have been several reports where the doping level decreased with increasing temperature.^[^ ^10^ ^]^ In contrast, the resulting charge transfer state for F4TCNQ/[Cs^+^TFSI^−^] (anion exchange), [TPA^• +^TFSI^−^], and [NO^• +^SbF_6_^−^] consists of ion pairs with closed‐shell ions (TFSI^−^ or SbF_6_^−^), which prevents the back‐transfer. Therefore, the surface doping state can be protected in conjunction with the closed‐shell anions. The most stable doping state was observed for samples having ion pairs with TFSI^−^, presumably because of the excellent hydrophobicity of TFSI^−^compared to that of SbF_6_^−^. The hydrophobicity and chemical stability of anions are thus both important for improving thermal durability, and further improvement will be achieved by tuning the physicochemical properties of the anions.

### 2.3 Demonstration of Strain Sensor

**Figure 5:**
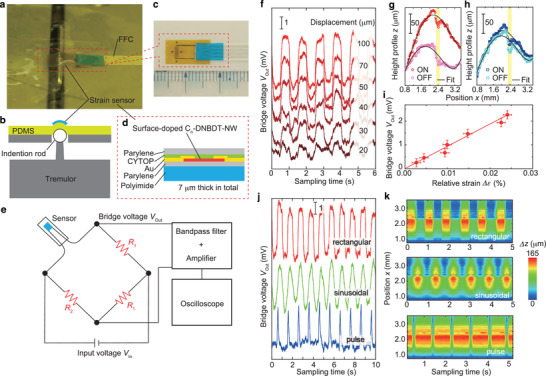
Demonstration of strain sensor. a) Photograph and b) schematic illustration of experimental setup. The strain sensor based on the surface‐doped C_8_–DNBDT thin films was mounted on the PDMS film. Tensile strain was applied to the strain sensor by pushing an indention rod into the PDMS film. c) Photograph and d) schematic illustration of the present strain sensor. The surface‐doped C_8_–DNBDT thin film was fabricated on a hybrid flexible substrate (parylene and polyimide). The resulting 7 µm thick strain sensor was connected electrically to the external Wheatstone bridge circuit shown in (e). f) Time domain profiles of output voltage *V*_out_ with respect to different displacements controlled by the indention rod. Surface height profiles monitored by a laser displacement meter under each applied displacement. As an example, the surface height profiles (*z*) with the displacement ON and OFF, which correspond to the indention rod being up and down, respectively, are shown in (g) 100 µm and (h) 20 µm. The height profiles were fit with a polynomial function (black curves), and were then converted to the applied strain, ϵ. The active area of the strain sensor was placed at *x* = 2.4 mm (highlighted in yellow). i) Dependence of the relative strain Δϵ, on the bridge voltage, *V*_out_. The error bars in both Δϵ (horizontal axis) and *V*_out_ (vertical axis) stem from compound errors that result from propagation of the uncertainties in fitting, and represent one standard deviation. j) Time domain profiles of *V*_out_ for various signal shapes (rectangular, sinusoidal, and pulse), and k) corresponding surface displacement, Δ*z*.

The excellent charge conduction, high strain sensitivity, and environmental stability realized with the surface‐doped C_8_–DNBDT may allow for the ideal production of internet of things (IoT) sensor devices. Sensors composed of a simple resistor would be advantageous in terms of reduced electronic noise. Electronic noise, which is a temporal fluctuation in electronic current, appears in any electronic circuit, and its magnitude in a resistor is proportional to the square root of its resistance. Therefore, the use of a resistor instead of a transistor and capacitor as a sensor could facilitate noise management in an electronic circuit. **Figure** **5** shows a demonstration of a strain sensor composed of surface doped OSCs. Here, F4TCNQ/[Cs^+^TFSI^−^] (anion exchange) was used as a dopant. Thin film strain sensor devices with a relatively low resistance of 40 kΩ were successfully fabricated on a thin polyimide film with a 1‐µm parylene layer (Figure 5a–d), where the total thickness was 7 µm. The strain sensor was mounted on a polydimethylsiloxane (PDMS) film, and the tensile strain was applied to the PDMS by pushing an indention rod. The output voltage (*V*_out_) was measured using an external Wheatstone bridge circuit, bandpass amplifier, and oscilloscope (Figure 5e), which is a standard configuration frequently used in commercially available strain sensing systems. Figure 5f shows time domain profiles for *V*_out_ for different displacements controlled by the indention rod. To accurately determine the changes in surface curvature and relative strain Δϵ, a laser displacement meter was used to monitor height profiles (*z*) as a function of the horizontal position (*x*, shown in Figure 5g,h). As a result, *V*_out_ was determined to increase linearly with Δϵ (Figure 5i), from which the GF was found to be approximately 20, which is consistent with that experimentally observed in Figure 4c. Based on the noise level in the present system, tensile strain changes of approximately 5× 10^−3^% (50 *μ*strain) are detectable. Time domain profiles of *V*_out_ also confirmed that various signal shapes are detectable (Figure 5j): the input displacement of rectangular, sinusoidal, and pulse signals (Figure 5k) could be clearly resolved. The overall results confirmed that the excellent electronic properties at the surface of the OSC by doping can be extended in device applications.

## 3 Conclusion

In conclusion, we have successfully presented a general strategy to functionalize the surface of single‐crystal OSCs. Structural and electrical measurements revealed that an integer number of charge transfers occurs only at the surface of the OSCs, which results in the formation of a coherent, 2D electronic system. The conductive nanosheet with a relatively large sheet conductivity of 10 *μ*S and a strain sensitivity with a GF up to 17 can be realized, while the original crystal structure is preserved after doping, and the carrier momentum relaxation is still dominated by phonon scattering, that is, the influence of ionized impurity scattering is negligible, even though a relatively high density of ionized impurities are in counter‐balance to charges on the polymer. Optimization and selection of the doping route and dopants allow for a highly stable conductive nanosheet that can be protected by inert closed‐shell anions. The present doping method to realize 2D nanosheets, as well as variation of dopants, is expected to serve as multifunctional platform for the production of various sensors.

## 4 Experimental Section

##### Surface Doping Methods

Organic crystalline films of C_8_–DNBDT were grown from a 0.02 wt% chlorothiophene solution by continuous edge casting. The pre‐cleaned substrate (125‐*μ*m‐thick polyethylene naphthalate (PEN) coated with 100‐nm‐thick parylene) was heated up to 60–62 °C, and moved with a shearing rate of 20 µm s^−1^ to give a bilayer single‐crystal film. Bilayer thin films were annealed at 80 °C in a vacuum overnight to remove residual solvent. The detailed conditions are given in refs. [22, 23, 25, 26]. Priori to the doping processes, Au electrodes were deposited through a shadow mask. Channel layers were then patterned by dry‐etching using an yttrium‐aluminum‐garnet laser (266 nm).

Molecular doping was performed simply by exposure of the solid‐state bilayer C_8_–DNBDT films to a solution of the dopant. Doping solutions were prepared in N_2_‐purged vials (commercially available, 7 mL). The combinations of solute (dopant) and solvents are summarized in Table 1. Solvents that are available to dissolve C_8_–DNBDT are limited to only acetonitrile (ACN) and *n*‐butyl acetate (*n*BA) because both are known to be orthogonal to C_8_–DNBDT. The dopant solutions were then removed by spinning the substrates,^[^ ^49^ ^]^ and the residual solvent was evaporated under a flow of N_2_. Doped *p*‐mTFF‐F3TSFI solution was prepared by first dissolving the solid form of the undoped mTFF‐TFSI‐Na (annealed for 1 h at 120 °C in a N_2_‐glovebox prior to dissolution) in anhydrous acetonitrile, 1.0 equiv. of NO^• +^SbF_6_^−^was then added to give the doped polymer solution. Excess ions and impurities were removed by precipitation with dimethyl carbonate and redissolution in acetonitrile to give *p*‐mTFF‐F3TSFI solution.

##### Characterization of Doping

Electrical measurements under compressive and tensile strain were conducted with the semiconductor parameter analyzer in conjunction with an in‐house‐built rig. The surface strain ϵ was calculated using the equation^[^ ^45^ ^]^ $\varepsilon =\frac{h_{s}}{2r}\times 100$, where *h*_s_ is the thickness of the substrate, which was 125 µm. Note that *h*_s_ should be the total thickness of the sample including the substrate and the OSC layer. However, the OSC layer was sufficiently thin; therefore, *h*_s_ can be approximated to the thickness of the PEN substrate. *r* is the curvature radius, which was measured directly with a laser displacement meter (Keyence LJ‐V7000). Compressive and tensile strains were applied repeatedly to eliminate any extrinsic drift/background effects. Ultra‐thin strain sensor devices were fabricated in a similar manner, except that polyimide (5 µm thick, Toyobo) was coated with an additional insulating polymer parylene layer (1 *μ*m thick) as the substrate, and a multi‐layer of fluorinated polymer CYTOP (200 nm)/parylene (1 µm) was employed as a protection layer. The polyimide film was first deposited and supported on a glass substrate. After all of the device fabrication processes, the completed strain sensor device was peeled off from the glass substrate via a laser lift‐off method.^[^ ^50^ ^]^ The strain sensor was then mounted onto a PDMS film. Tensile strain was applied to the strain sensor by pushing an indention rod into the PDMS film, where the indention rod was controlled by a tremulor (Asahi Seisakusyo, WaveMaker01). The magnitude of applied strain was estimated by monitoring the dynamic changes in the surface curvature with a laser displacement meter (Keyence LJ‐V7000). The strain sensor was connected electrically to an external Wheatstone bridge circuit by a flexible flat cable. The output voltage (bridge voltage *V*_out_), was then amplified and filtered by a bandpass filter (Stanford Instrument, SR560), and monitored with an oscilloscope (Tektronics, MDO3014).

## Conflict of Interest

The authors declare no conflict of interest.

## Author Contributions

S.W. conceived and designed the experiments, and analyzed all the data. K.Y., Y.Y., R.H., M.S., and H.N. fabricated and optimized all devices. R.H., and S.K., performed single‐crystal XRD measurements. C.G.T., L.‐L.C. and P.K.H.H. synthesized and provided the processing conditions of the self‐compensated doped polymer. T.O. synthesized and purified C_8_–DNBDT. S.W. wrote the manuscript with significant input from J.T. S.W. and J.T. supervised this work. All authors discussed the results and reviewed the manuscript.
